# 2-Phenyl­anilinium dihydrogen phosphate

**DOI:** 10.1107/S160053681003535X

**Published:** 2010-09-11

**Authors:** Mohamed Lahbib Mrad, Salah Ammar, Valeria Ferretti, Mohamed Rzaigui, Cherif Ben Nasr

**Affiliations:** a Laboratoire de Chimie des Matériaux, Faculté des Sciences de Bizerte, 7021 Zarzouna, Tunisia; b Faculté des Sciences de Gabes, Gabes, Tunisia; c Chemistry Department and Centro di Strutturistica Diffrattometrica, University of Ferrara, Via L. Borsari 46, I-44121 Ferrara, Italy; d Matériaux, Faculté des Sciences de Bizerte, 7021 Zarzouna, Tunisia

## Abstract

In the crystal structure of the title compound, C_12_H_12_N^+^·H_2_PO_4_^−^, the dihydrogen phosphate anions and the 2-phenyl­anilinium cations are associated *via* O—H⋯O and N—H⋯O hydrogen bonds so as to build inorganic layers around the *x* = 1/2 plane. The organic entities are anchored between these layers through C—H⋯O hydrogen bonds, forming a three-dimensional infinite network. The dihedral angle between the aromatic rings is 44.7 (4)°.

## Related literature

For related inorganic-organic materials, see: Mrad *et al.* (2006 ▶); Oueslati *et al.* (2009 ▶). For the organization of inorganic networks, see: Baoub & Jouini (1998 ▶). For the geometry around the P atom, see: Kefi *et al.* (2007 ▶); Oueslati & Ben Nasr (2006 ▶).
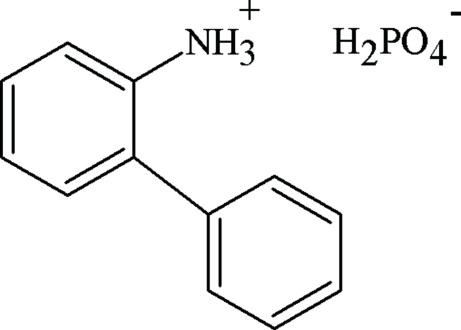


## Experimental

### 

#### Crystal data

C_12_H_12_N^+^·H_2_PO_4_^−^
*M* *_r_* = 267.21
Monoclinic, 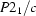

*a* = 15.4580 (4) Å
*b* = 4.7422 (1) Å
*c* = 18.4765 (6) Å
β = 112.008 (1)°
*V* = 1255.72 (6) Å^3^
*Z* = 4
Mo *K*α radiation
μ = 0.23 mm^−1^
*T* = 295 K
0.26 × 0.23 × 0.11 mm

#### Data collection

Nonius KappaCCD diffractometer
11492 measured reflections
3628 independent reflections
2106 reflections with *I* > 2σ(*I*)
*R*_int_ = 0.089

#### Refinement

*R*[*F* ^2^ > 2σ(*F* ^2^)] = 0.054
*wR*(*F* ^2^) = 0.137
*S* = 1.04
3628 reflections
183 parameters
5 restraints
H atoms treated by a mixture of independent and constrained refinement
Δρ_max_ = 0.26 e Å^−3^
Δρ_min_ = −0.36 e Å^−3^

### 

Data collection: *KappaCCD Server Software* (Nonius, 1997 ▶); cell refinement: *DENZO-SMN* (Otwinowski & Minor, 1997 ▶); data reduction: *DENZO-SMN*; program(s) used to solve structure: *SIR97* (Altomare *et al.*, 1999 ▶); program(s) used to refine structure: *SHELXL97* (Sheldrick, 2008 ▶); molecular graphics: *ORTEP-3* (Farrugia, 1997 ▶); software used to prepare material for publication: *WinGX* (Farrugia, 1999 ▶).

## Supplementary Material

Crystal structure: contains datablocks global, I. DOI: 10.1107/S160053681003535X/bg2358sup1.cif

Structure factors: contains datablocks I. DOI: 10.1107/S160053681003535X/bg2358Isup2.hkl

Additional supplementary materials:  crystallographic information; 3D view; checkCIF report

## Figures and Tables

**Table 1:** Hydrogen-bond geometry (Å, °)

*D*—H⋯*A*	*D*—H	H⋯*A*	*D*⋯*A*	*D*—H⋯*A*
N1—H1⋯O1^i^	0.90 (2)	2.07 (2)	2.950 (2)	167 (3)
N1—H2⋯O3	0.92 (3)	1.90 (3)	2.818 (3)	171 (2)
N1—H3⋯O1^ii^	0.90 (3)	1.83 (2)	2.727 (2)	173 (2)
O2—H13⋯O3^iii^	0.85 (2)	1.66 (2)	2.500 (2)	171 (3)
O4—H14⋯O4^iv^	0.80 (3)	2.00 (3)	2.797 (3)	172 (5)
C2—H4⋯O2	0.93	2.51	3.376 (3)	156
C9—H9⋯O2^v^	0.93	2.56	3.407 (3)	151

Symmetry codes: (i) 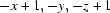; (ii) 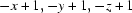; (iii) 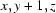; (iv) 
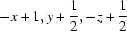
; (v) 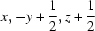.

**Fig. 1.**
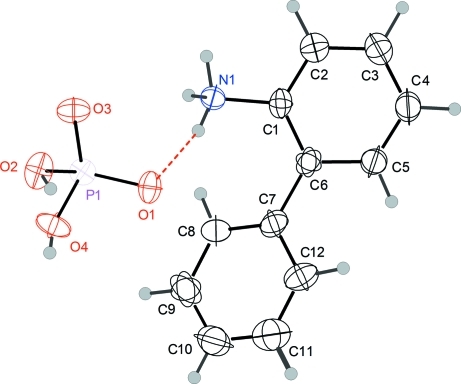
A view of (I), showing 50% probability displacement ellipsoids and arbitrary spheres for the H atoms.

**Fig. 2.**
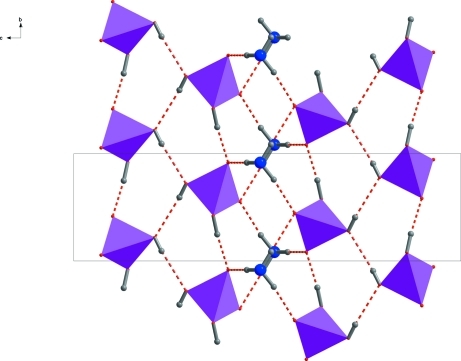
The packing diagram of the compound viewed down the b axis. PO4 is given in the tetrahedral representation. Hydrogen bonds are shown as dashed lines.

**Fig. 3.**
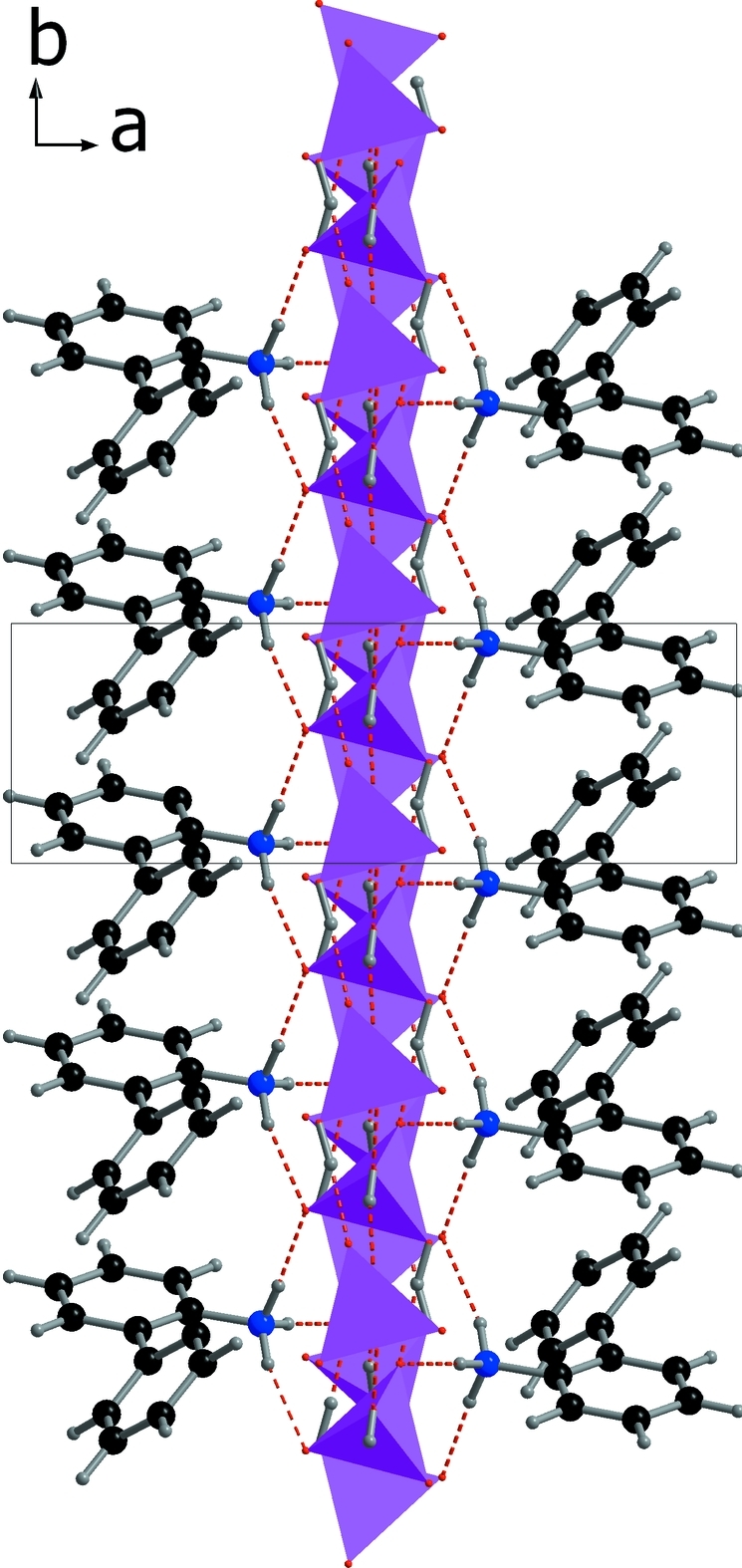
The packing diagram of the compound viewed down the a axis. PO4 is given in the tetrahedral representation. Hydrogen bonds are shown as dashed lines.

## supplementary crystallographic information

### Comment

During the systematic investigation of interaction between monophosphoric acid with organic molecules, numerous structures of monophosphates with organic cations have been described (Oueslati *et al.*, 2009; Mrad *et al.*, 2OO6). These structures are based on various one-, two-, or three-dimensional inorganic network depending on the nature and the shape of the organic molecule (Baoub and Jouini, 1998). Hydrogen bonds take part to the stability and the cohesion of the corresponding compounds. We report, in this work, the chemical preparation and the structural investigation of a new 2-Phenylanilinium dihydrogenomonophosphate. As shown in Fig. 1, the crystal structure of the title compound (I), consists of one phosphate anion and one organic cation. The H_2_PO_4_^-^ entities and the NH_3_^+^ ammonium catins have a layered organization around *x* = 1/2 plane (Fig. 2). Fig. 3 represents a projection of such layer. It shows that the H_2_PO_4_^-^ groups are connected by strong hydrogen bonds to form infinite chains in the b-direction of composition (H_2_PO_4_^-^)_n_^n-^. Each chain is interconnected, on one hand, to another one by means of O—H···O hydrogen bonds and, on the other hand, to NH_3_^+^ groups *via* N—H···O hydrogen bonds as to build an inorganic layer. The organic groups are anchored onto successive inorganic layers throw C—H···O hydrogen bonds (Fig. 2).

With regards to the H_2_PO_4_^-^ geometrical features, we remark the existence of two types of P···O distances. The longest ones (1.557 (2) and 1.595 (2) Å) correspond to P—OH groups, the shortest ones (1.494 (2) and 1.504 (2) Å) corresponding to classical P···O bonds. The average of the P···O distances and the O—P—O angles are 1.537 (2) Å and 109.35 (10)°, respectively. They agree perfectly with that generally observed for anions in other phosphates (Kefi *et al.*, 2OO7). The O—P—O angles spread in the range 104.87 (10) and 115.19 (10)°. This distortion from the ideal tetrahedral value has been regularly noted in other organic phosphates (Oueslati and Ben Nasr, 2006). The larger mean value of 112.70 (10)°, with a range of 110.74 (11)–115.19 (10)°, corresponds to the O—P—O angle. The smaller one 106.00 (10)°, with a range of 104.88 (10)–107.89 (10)°, is related to the HO—P—OH angle. All these geometrical parameters are in full agreement with those observed in such anions in other organic dihydrogenomonophosphates (Oueslati and Ben Nasr, 2006). However, the P—P distance between H_2_PO_4_ tetrahedra: 4.742 (2) ° is slightly shorter than that observed in NH_3_(CH_2_)_4_NH_3_HPO_4_.H_2_O (Baoub and Jouini, 1998) [5.575 (1) °], which is probably due to the presence of two acidic hydrogen atoms on the PO_4_ leading to the formation of strong hydrogen bonds. Furthermore, the short P—P distance is in favour of the general formation of [H_2_PO_4_^-^]_n_^n-^ polyanions in the crystal structure, but not to the individualization of the H_2_PO_4_^-^ groups (Oueslati and Ben Nasr, 2006).

### Experimental

Crystals of the title compound have been prepared in a Petri dish by adding 50 mmol of concentrated orthophosphoric acid (Fluka, 85%, d = 1.7) to 25 mmol of 2-phenylaniline (Acros) dissolved in ethanol. After agitation, the resulting solution has been slowly evaporated at room temperature until the formation of single crystals suitable for X-ray structure analysis and remained stable under normal conditions of temperature and humidity.

### Refinement

Hydrogen atoms bound to N and O atoms were located in the Difference Fourier map and refined with restraints on the bond length [0.87 (2) and 0.84 (2) for N—H and O—H, respectively] ; the remaining H atoms were given calculated positions (C–H: 0.93Å) . In all cases riding displacement factors were used, with a multiplication factor of 1.2.

### Crystal data

**Table:**

C_12_H_12_N^+^·H_2_PO_4_^−^	*F*(000) = 560
*M**_r_* = 267.21	*D*_x_ = 1.413 Mg m^−^^3^
Monoclinic, *P*2_1_/*c*	Mo *K*α radiation, λ = 0.71073 Å
Hall symbol: -P 2ybc	Cell parameters from 11492 reflections
*a* = 15.4580 (4) Å	θ = 5.0–30.0°
*b* = 4.7422 (1) Å	µ = 0.23 mm^−^^1^
*c* = 18.4765 (6) Å	*T* = 295 K
β = 112.008 (1)°	Plate, colourless
*V* = 1255.72 (6) Å^3^	0.26 × 0.23 × 0.11 mm
*Z* = 4	

### Data collection

**Table:**

Nonius KappaCCD diffractometer	2106 reflections with *I* > 2σ(*I*)
Radiation source: fine-focus sealed tube	*R*_int_ = 0.089
graphite	θ_max_ = 30.2°, θ_min_ = 5.3°
φ and ω scans	*h* = −21→21
11492 measured reflections	*k* = −6→6
3628 independent reflections	*l* = −25→24

### Refinement

**Table:**

Refinement on *F*^2^	5 restraints
Least-squares matrix: full	H atoms treated by a mixture of independent and constrained refinement
*R*[*F*^2^ > 2σ(*F*^2^)] = 0.054	*w* = 1/[σ^2^(*F*_o_^2^) + (0.0566*P*)^2^ + 0.2547*P*] where *P* = (*F*_o_^2^ + 2*F*_c_^2^)/3
*wR*(*F*^2^) = 0.137	(Δ/σ)_max_ = 0.001
*S* = 1.04	Δρ_max_ = 0.26 e Å^−^^3^
3628 reflections	Δρ_min_ = −0.36 e Å^−^^3^
183 parameters	

### Special details

**Table:**

Geometry. Bond distances, angles *etc*. have been calculated using the rounded fractional coordinates. All su's are estimated from the variances of the (full) variance-covariance matrix. The cell e.s.d.'s are taken into account in the estimation of distances, angles and torsion angles

### Fractional atomic coordinates and isotropic or equivalent isotropic displacement parameters (Å^2^)

**Table:**

	*x*	*y*	*z*	*U*_iso_*/*U*_eq_	
P1	0.50007 (4)	0.34843 (11)	0.37476 (3)	0.03014 (17)	
O1	0.59397 (10)	0.4449 (3)	0.42905 (9)	0.0387 (4)	
O2	0.42429 (11)	0.5807 (3)	0.36048 (11)	0.0442 (4)	
H13	0.441 (2)	0.751 (4)	0.3706 (19)	0.078 (11)*	
O3	0.46470 (12)	0.0835 (3)	0.39908 (10)	0.0391 (4)	
O4	0.50106 (13)	0.2818 (4)	0.29045 (10)	0.0447 (4)	
H14	0.506 (3)	0.425 (6)	0.269 (2)	0.098 (14)*	
N1	0.34451 (13)	0.0832 (4)	0.48207 (12)	0.0338 (4)	
H1	0.3538 (18)	−0.079 (4)	0.5088 (15)	0.049 (8)*	
H2	0.3783 (15)	0.083 (5)	0.4503 (13)	0.039 (7)*	
H3	0.3664 (19)	0.231 (5)	0.5148 (15)	0.058 (8)*	
C1	0.24645 (15)	0.1339 (5)	0.43239 (13)	0.0352 (5)	
C2	0.22923 (18)	0.2634 (6)	0.36147 (15)	0.0492 (6)	
H4	0.2788	0.316	0.3474	0.059*	
C3	0.13871 (19)	0.3153 (7)	0.31125 (16)	0.0575 (7)	
H5	0.1269	0.402	0.2633	0.069*	
C4	0.06575 (18)	0.2368 (7)	0.33299 (16)	0.0548 (7)	
H6	0.0045	0.2697	0.2995	0.066*	
C5	0.08346 (17)	0.1110 (6)	0.40363 (15)	0.0472 (6)	
H7	0.0335	0.0599	0.4173	0.057*	
C6	0.17466 (16)	0.0565 (5)	0.45629 (14)	0.0378 (5)	
C7	0.18893 (16)	−0.0716 (5)	0.53375 (14)	0.0400 (5)	
C8	0.25463 (17)	0.0340 (6)	0.60238 (14)	0.0458 (6)	
H8	0.2927	0.1833	0.6005	0.055*	
C9	0.2639 (2)	−0.0821 (7)	0.67409 (16)	0.0601 (8)	
H9	0.3089	−0.0127	0.7198	0.072*	
C10	0.2068 (2)	−0.2983 (7)	0.67742 (19)	0.0642 (8)	
H10	0.2129	−0.3751	0.7254	0.077*	
C11	0.1406 (2)	−0.4018 (7)	0.6100 (2)	0.0659 (9)	
H11	0.1015	−0.547	0.6126	0.079*	
C12	0.13193 (19)	−0.2921 (6)	0.53876 (17)	0.0508 (6)	
H12	0.0875	−0.3659	0.4934	0.061*	

### Atomic displacement parameters (Å^2^)

**Table:**

	*U*^11^	*U*^22^	*U*^33^	*U*^12^	*U*^13^	*U*^23^
P1	0.0349 (3)	0.0253 (3)	0.0303 (3)	0.0000 (2)	0.0123 (2)	−0.0027 (2)
O1	0.0337 (8)	0.0377 (8)	0.0398 (9)	−0.0021 (6)	0.0080 (7)	−0.0056 (7)
O2	0.0355 (9)	0.0292 (9)	0.0612 (12)	0.0030 (7)	0.0104 (8)	−0.0065 (7)
O3	0.0502 (9)	0.0277 (7)	0.0448 (10)	−0.0024 (7)	0.0239 (8)	−0.0012 (6)
O4	0.0651 (11)	0.0398 (9)	0.0326 (9)	−0.0012 (8)	0.0223 (8)	−0.0027 (7)
N1	0.0291 (9)	0.0379 (11)	0.0336 (11)	−0.0007 (8)	0.0108 (8)	−0.0024 (8)
C1	0.0307 (10)	0.0397 (12)	0.0310 (11)	0.0007 (9)	0.0068 (9)	−0.0052 (9)
C2	0.0394 (13)	0.0655 (17)	0.0417 (15)	−0.0008 (12)	0.0141 (11)	0.0059 (12)
C3	0.0472 (15)	0.079 (2)	0.0399 (15)	0.0082 (14)	0.0085 (12)	0.0140 (14)
C4	0.0349 (13)	0.0761 (18)	0.0423 (15)	0.0040 (13)	0.0016 (11)	0.0018 (13)
C5	0.0307 (12)	0.0621 (16)	0.0456 (14)	−0.0016 (11)	0.0106 (10)	−0.0032 (12)
C6	0.0342 (11)	0.0428 (12)	0.0358 (12)	−0.0021 (10)	0.0122 (10)	−0.0060 (10)
C7	0.0359 (12)	0.0459 (13)	0.0411 (13)	0.0055 (10)	0.0175 (10)	−0.0019 (10)
C8	0.0399 (12)	0.0604 (15)	0.0390 (14)	0.0013 (12)	0.0169 (11)	−0.0035 (12)
C9	0.0544 (16)	0.088 (2)	0.0389 (15)	0.0158 (15)	0.0186 (13)	0.0020 (14)
C10	0.071 (2)	0.077 (2)	0.0562 (19)	0.0182 (17)	0.0371 (16)	0.0226 (16)
C11	0.070 (2)	0.0591 (18)	0.084 (2)	0.0060 (15)	0.0460 (19)	0.0164 (16)
C12	0.0467 (14)	0.0524 (15)	0.0586 (17)	−0.0005 (12)	0.0258 (13)	−0.0008 (13)

### Geometric parameters (Å, °)

**Table:**

P1—O1	1.4937 (16)	C4—C5	1.366 (4)
P1—O3	1.5045 (15)	C4—H6	0.93
P1—O2	1.5566 (16)	C5—C6	1.405 (3)
P1—O4	1.5952 (17)	C5—H7	0.93
O2—H13	0.844 (18)	C6—C7	1.493 (3)
O4—H14	0.807 (18)	C7—C8	1.388 (3)
N1—C1	1.468 (3)	C7—C12	1.393 (4)
N1—H1	0.895 (17)	C8—C9	1.392 (4)
N1—H2	0.921 (16)	C8—H8	0.93
N1—H3	0.905 (17)	C9—C10	1.368 (4)
C1—C2	1.380 (3)	C9—H9	0.93
C1—C6	1.389 (3)	C10—C11	1.372 (5)
C2—C3	1.381 (4)	C10—H10	0.93
C2—H4	0.93	C11—C12	1.375 (4)
C3—C4	1.383 (4)	C11—H11	0.93
C3—H5	0.93	C12—H12	0.93
			
O1—P1—O3	115.19 (10)	C3—C4—H6	119.9
O1—P1—O2	112.16 (9)	C4—C5—C6	122.1 (2)
O3—P1—O2	107.87 (10)	C4—C5—H7	118.9
O1—P1—O4	110.73 (10)	C6—C5—H7	118.9
O3—P1—O4	105.30 (9)	C1—C6—C5	116.4 (2)
O2—P1—O4	104.88 (10)	C1—C6—C7	124.3 (2)
P1—O2—H13	120 (2)	C5—C6—C7	119.2 (2)
P1—O4—H14	111 (3)	C8—C7—C12	118.4 (2)
C1—N1—H1	113.6 (18)	C8—C7—C6	121.6 (2)
C1—N1—H2	107.5 (15)	C12—C7—C6	120.0 (2)
H1—N1—H2	110 (2)	C9—C8—C7	120.4 (3)
C1—N1—H3	109.3 (18)	C9—C8—H8	119.8
H1—N1—H3	111 (2)	C7—C8—H8	119.8
H2—N1—H3	105 (2)	C10—C9—C8	120.1 (3)
C2—C1—C6	121.8 (2)	C10—C9—H9	120
C2—C1—N1	117.0 (2)	C8—C9—H9	120
C6—C1—N1	121.3 (2)	C11—C10—C9	120.1 (3)
C1—C2—C3	120.2 (2)	C11—C10—H10	120
C1—C2—H4	119.9	C9—C10—H10	120
C3—C2—H4	119.9	C10—C11—C12	120.4 (3)
C2—C3—C4	119.2 (3)	C10—C11—H11	119.8
C2—C3—H5	120.4	C12—C11—H11	119.8
C4—C3—H5	120.4	C11—C12—C7	120.7 (3)
C5—C4—C3	120.1 (2)	C11—C12—H12	119.7
C5—C4—H6	119.9	C7—C12—H12	119.7

### Hydrogen-bond geometry (Å, °)

**Table:**

*D*—H···*A*	*D*—H	H···*A*	*D*···*A*	*D*—H···*A*
N1—H1···O1^i^	0.90 (2)	2.07 (2)	2.950 (2)	167 (3)
N1—H2···O3	0.92 (3)	1.90 (3)	2.818 (3)	171 (2)
N1—H3···O1^ii^	0.90 (3)	1.83 (2)	2.727 (2)	173 (2)
O2—H13···O3^iii^	0.85 (2)	1.66 (2)	2.500 (2)	171 (3)
O4—H14···O4^iv^	0.80 (3)	2.00 (3)	2.797 (3)	172 (5)
C2—H4···O2	0.93	2.51	3.376 (3)	156
C9—H9···O2^v^	0.93	2.56	3.407 (3)	151

Symmetry codes: (i) −*x*+1, −*y*, −*z*+1; (ii) −*x*+1, −*y*+1, −*z*+1; (iii) *x*, *y*+1, *z*; (iv) −*x*+1, *y*+1/2, −*z*+1/2; (v) *x*, −*y*+1/2, *z*+1/2.
